# BLV-CoCoMo Dual qPCR Assay Targeting LTR Region for Quantifying Bovine Leukemia Virus: Comparison with Multiplex Real-Time qPCR Assay Targeting *pol* Region

**DOI:** 10.3390/pathogens13121111

**Published:** 2024-12-16

**Authors:** Sonoko Watanuki, Aronggaowa Bao, Etsuko Saitou, Kazuyuki Shoji, Masaki Izawa, Mitsuaki Okami, Yasunobu Matsumoto, Yoko Aida

**Affiliations:** 1 Laboratory of Global Infectious Diseases Control Science, Graduate School of Agricultural and Life Sciences, The University of Tokyo, 1-1-1 Yayoi, Bunkyo-ku, Tokyo 113-8657, Japan; watanuki-sonoko228@g.ecc.u-tokyo.ac.jp (S.W.);; 2 Hyogo Prefectural Awaji Meat Inspection Center, 49-18 Shitoorinagata, Minamiawaji 656-0152, Japan; 3 Molecular Diagnosis Division, Nippon Gene Co., Ltd., 2-8-16 Toiya-machi, Toyama 930-0834, Japan; 4 Laboratory of Global Animal Resource Science, Graduate School of Agricultural and Life Sciences, The University of Tokyo, 1-1-1 Yayoi, Bunkyo-ku, Tokyo 113-8657, Japan

**Keywords:** bovine leukemia virus (BLV), proviral load (PVL), quantitative real-time PCR (qPCR), duplex, long terminal repeat (LTR), *pol* gene, commercial kit

## Abstract

The proviral load (PVL) of the bovine leukemia virus (BLV) is a useful index for estimating disease progression and transmission risk. Real-time quantitative PCR techniques are widely used for PVL quantification. We previously developed a dual-target detection method, the “Liquid Dual-CoCoMo assay”, that uses the coordination of common motif (CoCoMo) degenerate primers. This method can detect two genes simultaneously using a FAM-labeled minor groove binder (MGB) probe for the BLV long terminal repeat (LTR) region and a VIC-labeled MGB probe for the *BoLA-DRA* gene. In this study, we evaluated the diagnostic and analytical performance of the Dual-CoCoMo assay targeting the LTR region by comparing its performance against the commercially available Takara multiplex assay targeting the *pol* region. The diagnostic sensitivity and specificity of the Liquid Dual-CoCoMo assay based on the diagnostic results of the ELISA or original Single-CoCoMo qPCR were higher than those of the Takara multiplex assay. Furthermore, using a BLV molecular clone, the analytical sensitivity of our assay was higher than that of the Takara multiplex assay. Our results provide the first evidence that the diagnostic and analytical performances of the Liquid Dual-CoCoMo assay are better than those of commercially available multiplex assays that target the *pol* region.

## 1. Introduction

Bovine leukemia virus (BLV), which belongs to the *Deltaretrovirus* genus of the *Retroviridae* family, is the etiological agent of enzootic bovine leukosis (EBL) and is closely related to human T-cell leukemia virus types 1 and 2 [1,2,3,4,5]. Approximately 70% of cattle infected with BLV show no clinical symptoms, whereas 30% develop persistent lymphocytosis, and 2–5% of BLV-infected cattle form B-cell lymphoma after a long latency period [1,6,7,8,9,10]. BLV infection is highly prevalent in many countries, except for some European countries and Oceania [11], causing serious economic damage due to reduced milk production [12,13,14,15], carcass weight loss [16], fertility [17], and longevity [14,18,19]. In addition, import and export regulations have been implemented for BLV-infected animals in certain countries and regions [11], yielding incalculable losses worldwide.

In Japan, a nationwide survey of BLV infection showed that 40.9% of dairy and 28.7% of beef-breeding cattle were infected with BLV [20]. However, there are no effective treatments or commercially available vaccines for preventing this disease [21]. Under these circumstances, the main approach is segregating or culling animals infected with BLV from herds by accurately testing for BLV infection and preventing the spread of new infections [22]. One of the diagnostic methods used to determine whether a cow is infected is the BLV provirus detection method based on the polymerase chain reaction (PCR) [23,24,25,26,27,28,29,30,31]. The BLV provirus remains integrated into the host genome with or without antibodies [32] and can be amplified after a period of latency [32,33]. Furthermore, the BLV proviral load (PVL), that is, the number of copies of the BLV provirus, is a useful diagnostic marker for implementing prevention or eradication strategies against BLV because the PVL is associated with disease progression [34,35,36,37] and transmission risk [34,38,39,40]. Thus, real-time quantitative PCR (qPCR) for quantifying BLV PVL is used worldwide.

We previously developed a TaqMan-based real-time qPCR assay, the "BLV-CoCoMo-qPCR" assay (Single-CoCoMo assay), using the coordination of common motifs (CoCoMo) primers, which were designed for both known and novel BLV variants in animals naturally infected with BLV [34,41]. The complete genome of BLV comprises 8714 nucleotides, including the structural and enzymatic *gag*, *pro*, *pol*, and *env* essential genes, non-structural *tax*, *rex*, *R3*, and *G4* genes, and two identical long terminal repeats (LTRs) [1]. Notably, the Single-CoCoMo assay has enhanced sensitivity compared to that of other methods because it detects the BLV LTR region, which is present at two copies per provirus [34]. In addition, this assay indicates the BLV PVL as the number of copies per cell by amplifying it in parallel with the bovine leukocyte antigen *(BoLA)-DRA* gene as a single-copy host gene and normalizing viral genomic DNA [34]. Furthermore, a modified version, “BLV-CoCoMo-qPCR-2”, which uses optimized degenerate primers and PCR conditions, as well as a reconstructed standard plasmid, was developed in 2015 [42]. Moreover, by improving the BLV-CoCoMo-qPCR-2 assay, we developed a simple and user-friendly dual-target detection qPCR assay called “BLV-CoCoMo Dual qPCR” assay (Liquid Dual-CoCoMo assay), which detects two genes simultaneously in the same well using a 6-carboxyfluorescein (FAM)-labeled minor groove binder (MGB) probe for the BLV LTR gene and a VIC-labeled MGB probe for the *BoLA-DRA* gene as internal control [43]. The report showed that the Liquid Dual-CoCoMo assay maintained the sensitivity and reproducibility of the Single-CoCoMo assay and can be used for measuring BLV PVLs, similar to the Single-CoCoMo assay [43].

The target regions for BLV provirus detection described in the World Organization for Animal Health (WOAH) Terrestrial Manual are the *env* and *pol* genes in the BLV proviral genome, including the *gag*, *pro*, *pol*, *env*, *tax*, *rex*, *R3*, *G4,* and LTR regions. Detecting these two regions is a standard procedure in the international beef trade in many countries [44,45,46,47,48,49,50]. In the USA, there is commercially available SS1 BLV proviral load qPCR multiplex assay (AntelBio^TM^ *STRATA-G*^TM^ BLV PCR) (CentralStar Cooperative, Lansing, MI, USA) composed of three TaqMan primer/probe assays that detect the BLV *pol* gene, *bovine β-Actin*, and an internal amplification control [51,52,53,54]. In addition, commercially available PCR reagents to detect the BLV *pol* gene are available in Korea (https://www.mediandiagnostics.com/en/22_view?idx=57; accessed on 2 September 2024). However, we previously showed that the Single-CoCoMo assay targeting the LTR region, which is present in two copies per provirus, had the highest analytical sensitivity compared to that of the TaqMan MGB assay developed by Lew et al. targeting the *pol* region, which is present in a single copy per provirus [45], and compared to that of the Cycleave PCR BLV detection kit (Takara-Cycleave assay) targeting the *tax* region, which is present in a single copy per provirus [41]. Furthermore, Yoneyama et al. demonstrated that the Single-CoCoMo assay targeting the LTR and Takara multiplex assay targeting the *pol* region, which is a dual-target detection system that detects target genes and internal controls simultaneously, are better than the Takara-Cycleave assay targeting the *tax* region [46]. However, no studies have compared the performance of the Liquid Dual-CoCoMo assay targeting the LTR region with methods targeting other regions to measure BLV PVLs.

In Japan, there are three commercially available real-time qPCR kits for BLV PVLs: (i) the Single-CoCoMo assay (Nippon Gene Co., Ltd., Toyama, Japan) [34,37,39,41,42,55,56,57,58,59,60], (ii) the Liquid Dual-CoCoMo assay targeting the BLV LTR region and *BoLA-DRA* gene as the internal control (Nippon Gene Co. Ltd.) [43], and (iii) the BLV multiplex qPCR detection kit (Takara multiplex assay) targeting the *pol* and *RPPH1* genes as the internal control (Takara Bio Inc., Otsu, Japan) [46], which is routinely used for measuring the BLV PVL. In this study, we compared the diagnostic and analytical performances of the Liquid Dual-CoCoMo and Takara multiplex assays, which target different gene regions and are commercially available in Japan, by measuring the BLV PVL in field samples and detecting a BLV molecular clone. The results of the present study enhance our understanding of the performance of commercial multiplex qPCR assays for BLV quantification and contribute significantly to prevention and eradication strategies against BLV.

## 2. Materials and Methods

### 2.1. Sample Collection, DNA Extraction, and Plasma Isolation

Blood samples were collected in tubes containing ethylenediaminetetraacetic acid (EDTA) from 214 dairy and beef cows from BLV-positive farms in Japan. Genomic DNA was extracted using the Wizard Genomic DNA Purification Kit (Promega Corporation, Tokyo, Japan) according to the manufacturer’s instructions and adjusted to 30 ng/µL for the qPCR assays. Portions of whole blood samples were used to separate plasma to detect anti-BLV antibodies. Veterinarians handled all the cattle in accordance with the guidelines of the University of Tokyo. This study was approved by the Animal Experiments Committee of the University of Tokyo (Approval Number: p22-2-030). 

### 2.2. Detection of anti-BLV gp51 Antibodies

Anti-BLV gp51 antibodies were detected using an anti-BLV antibody ELISA Kit (Nippon Gene), according to the manufacturer’s instructions.

### 2.3. Determination of the BLV Proviral Loads Using the BLV-CoCoMo-qPCR-2 “Single-CoCoMo Assay”

The BLV-CoCoMo-qPCR-2 assay (Nippon Gene) was performed to determine BLV PVLs using a THUNDERBIRD Probe qPCR Mix (Toyobo, Tokyo, Japan), as previously described [42]. Briefly, the BLV LTR region was amplified using the degenerate BLV CoCoMo primer mix and detected using a FAM-labeled BLV MGB probe included in the CoCoMo^TM^-BLV Primer/Probe kit (Nippon Gene). As an internal control, the *BoLA-DRA* gene was amplified using a DRA primer mix and detected using a FAM-labeled DRA MGB probe in the CoCoMo kit (Nippon Gene). The PCR conditions were 1 min at 95 °C, followed by 45 cycles at 95 °C for 15 s and 60 °C for 1 min. All the amplification steps were conducted on a Light Cycler^®^ 480 System II (Roche Diagnostics, Mannheim, Germany). Finally, the PVL was calculated using the following formula: (number of BLV LTR copies/number of *BoLA-DRA* copies) × 10^5^ cells. The BLV PVL was expressed as the number of copies per 10^5^ cells.

### 2.4. Determination of the BLV Proviral Loads Using the BLV-CoCoMo Liquid Dual qPCR Assay “Liquid Dual-CoCoMo Assay”

The BLV-CoCoMo Liquid Dual qPCR assay (Nippon Gene) was performed using a GeneAce Probe qPCR Mix II (Nippon Gene), as previously described [43]. Briefly, BLV PVLs were determined by simultaneously detecting two genes in the same well using FAM- and VIC-labeled MGB probes (Nippon Gene) for the BLV LTR and *BoLA-DRA* gene, respectively. The primers used for the Dual-CoCoMo assay were the degenerate BLV CoCoMo primer mix and a DRA primer mix (Nippon Gene). PCR conditions were 10 min at 95 °C, followed by 45 cycles at 95 °C for 15 s and 60 °C for 1 min. Samples showed a typical amplification curve and were defined as positive when the *BoLA-DRA* (control) and BLV LTR assays were positive, with threshold cycle (Ct) values of <45. The BLV PVL was indicated as the number of copies per 10^5^ cells.

### 2.5. Determination of the BLV Proviral Loads by Takara Multiplex Assay

The Takara multiplex assay was performed using the Bovine leukemia virus detection qPCR kit (RC201A) according to the manufacturer’s instructions (Takara Bio Inc., Otsu, Japan) on the Light Cycler^®^ 480 system II (Roche Diagnostics). This qPCR kit is a multiplex qPCR assay that simultaneously amplifies the BLV *pol* gene and *RPPH1* gene as an internal control. Briefly, the BLV *pol* and *RPPH1* genes were simultaneously amplified using primers targeting these genes and detected using FAM- and ROX-labeled probes for the BLV *pol* and *RPPH1* genes. The samples showed a typical amplification curve and were defined as positive when the *RPPH1* (control) and BLV *pol* assays were positive, with Ct values of <45. The PVL was calculated using the following formula: [number of BLV *pol* copies/(number of *RPPH1* copies/2)] × 10^5^ cells. The BLV PVL was expressed as the number of copies per 10^5^ cells.

### 2.6. PCR Amplification and Sequencing of BLV LTR Gene Fragments

The partial BLV LTR region from the cow that was negative in the Single-CoCoMo and Liquid Dual-CoCoMo assays but positive in the Takara multiplex assay was amplified using a previously described primer set [61]. Briefly, the PCR was performed in 20 µL reactions containing 0.6 µL of 10 µM CoMo-t1F primer (5′-ACGTCAGCTGCCAGAAAAGCTG-3′), 10 µM CoMo-t1R primer (5′-AGCCAGACGCCCTTGGAGCGCG-3′), 10 µL of 2×TaqMan Gene Expression Master Mix (Thermo Fisher, Tokyo, Japan), 3.8 µL of nuclease-free water, and 5 µL of genomic DNA adjusted to 30 ng/µL. The PCR conditions were 5 min at 95 °C, followed by 60 cycles at 95 °C for 15 s, 60 °C for 15 s, 72 °C for 30 s, and a final extension step of 7 min. The sequence analysis was performed by Fasmac Co., Ltd. using the CoMo-t2F (5′-CTGGTGACGGCAGCTGGTGGC-3′) and CoMo-t3R (5′-TAGAGCTCGCGGTGGTCTCAG-3′) [61]. The nucleotide sequences were aligned and identified using GENETYX version 10 (GENETYX Corporation, Tokyo, Japan).

### 2.7. Comparison of the Analytical Sensitivity of the Liquid Dual-CoCoMo and Takara Multiplex Assays

The analytical sensitivities of the Liquid Dual-CoCoMo and Takara multiplex assays were evaluated using an infectious full-length molecular clone of BLV (pBLV-IF2) [62,63]. The pBLV-IF2 was serially diluted two-fold in Tris-EDTA (TE) buffer to achieve provirus copy numbers from 25 to 0.78125. Each dilution was supplemented with 150 ng of genomic DNA from a BLV-uninfected cow and measured in quintuplicate, with the assays repeated twice. The analytical sensitivities of both assays were expressed as the percentage of successful amplifications.

### 2.8. Statistical Analysis

The paired *t*-test was used to compare the mean BLV PVL determined by the Liquid Dual-CoCoMo and Takara multiplex assays. Pearson’s correlation was used to evaluate the strength of the association of the quantitative values measured by the Liquid Dual-CoCoMo and Takara multiplex assays. All analyses were performed using R software (version 4.3.2). Cohen’s kappa statistics were calculated to assess the level of agreement between the results of each real-time qPCR assay and BLV infection diagnosis using EZR software [64] and interpreted as the following: almost perfect agreement (0.81–1.0), substantial agreement (0.61–0.80), moderate agreement (0.41–0.60), fair agreement (0.21–0.40), slight agreement (0.0–0.2), and no agreement (<0) [65].

## 3. Results

### 3.1. Diagnosis of BLV Infection Using Real-Time PCR and Serological Tests on 214 Field Cows

To determine the status of BLV infection in 214 field cows, we first performed a real-time PCR test with the original Single-CoCoMo assay targeting the BLV LTR region and a serological test with ELISA, which are routinely used to test BLV infection in Japan (Table 1). The results showed that 118 (55.1%) and 117 (54.7%) of 214 cows were positive in the ELISA and Single-CoCoMo assay, respectively. In contrast, 96 (44.9%) and 97 (45.3%) cows were negative in the ELISA and Single-CoCoMo assay, respectively. Next, we performed a duplex real-time PCR test with Liquid Dual-CoCoMo assay targeting the BLV LTR region and the Takara multiplex assay targeting the *pol* region to detect the BLV provirus (Table 1). The results showed that 115 (53.7%) and 111 (51.9%) of 214 cows were positive in the Liquid Dual-CoCoMo and Takara multiplex assays, respectively. On the contrary, 99 (46.3%) and 103 (48.1%) cows were negative in the Liquid Dual-CoCoMo and Takara multiplex assays, respectively.

By comparing the BLV detection results in ELISA and three qPCR assays, we found that 108 cows were positive in the ELISA and three qPCR assays, whereas 93 were negative in all tests (Table 2). Two cows (O1 and O2) were negative in three qPCR assays but positive in the ELISA. In addition, three cows (S40, O10, and O6) were positive for the BLV provirus in the qPCR assays but negative in ELISA. Among these three cows, one cow (S40) was positive in all three real-time PCR assays, one cow (O10) was positive only in the Single-CoCoMo assay, and one cow (O6) was positive only in the Takara multiplex assay.

### 3.2. Comparison of Results from Liquid Dual-CoCoMo and Takara Multiplex Assays

We compared the results determined by the Liquid Dual-CoCoMo and Takara multiplex assays using 121 cows, excluding 93 cows that were negative in all tests (Table 3). Among the 121 cows, 110 were positive in both multiplex qPCR assays, whereas five were negative in both assays. Five of the remaining six cows were positive in the Liquid Dual-CoCoMo assay but negative in the Takara multiplex assay. These five cows were also positive in the ELISA and Single-CoCoMo assay. In contrast, one cow (O6) tested negative in the Liquid Dual-CoCoMo assay but positive in the Takara multiplex assay. This cow was negative in the ELISA and Single-CoCoMo assay. Recently, three types of mutations were reported in the nucleobase of the probe region of the LTRs in the BLV-CoCoMo-qPCR-2 assay [61]. Therefore, we sequenced the LTR region of a cow (O6) to assess whether mutations were present in the probe region of the Liquid Dual-CoCoMo assay. We amplified the target region of this cow, and the partial sequence of the LTR region was sequenced and aligned with the FLK-BLV sequence (EF600696). The results showed no mutations in the LTR region (Figure S1).

**Figure 1:**
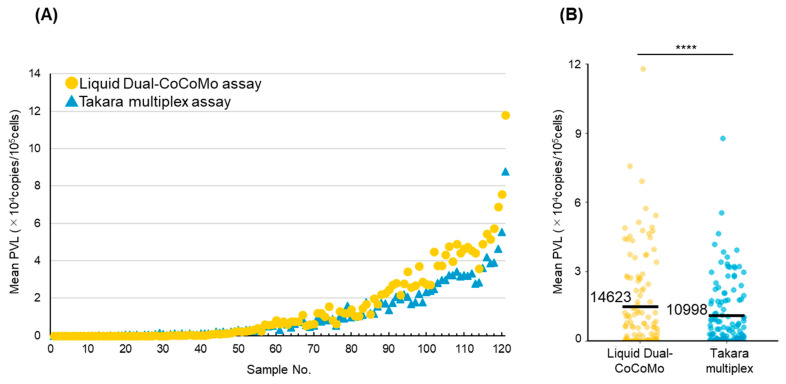
BLV PLVs of 121 cows determined by Liquid Dual-CoCoMo and Takara multiplex assays. (**A**) The PVLs of 121 cows, excluding BLV-uninfected cattle, were determined from those samples in duplicate using the Liquid Dual-CoCoMo (yellow circle) and Takara multiplex (blue triangle) assays. The PVL was expressed as the number of copies per 10^5^ cells. (**B**) Comparison of PVLs measured by Liquid Dual-CoCoMo (yellow) and Takara multiplex (blue) assays. The means are denoted by the black bars. The *p*-values were calculated using a paired *t*-test. Asterisks indicate significant differences (**** *p* < 0.0001).

The PVLs from 121 samples were measured using the Liquid Dual-CoCoMo and Takara multiplex assays. PVLs ranged from 0 to 117,881 copies per 10^5^ cells for the Liquid Dual-CoCoMo assay and from 0 to 87,645 copies per 10^5^ cells for the Takara multiplex assay (Figure 1A). The PVLs measured using the Liquid Dual-CoCoMo assay were higher than those measured using the Takara multiplex assay in field samples. As shown in Figure 1B, the mean PVLs measured by the Liquid Dual-CoCoMo assay (14,623 copies per 10^5^ cells) were significantly higher than those measured by the Takara multiplex assay (10,998 copies per 10^5^ cells) in 121 field samples (Figure 1B). 

**Figure 2:**
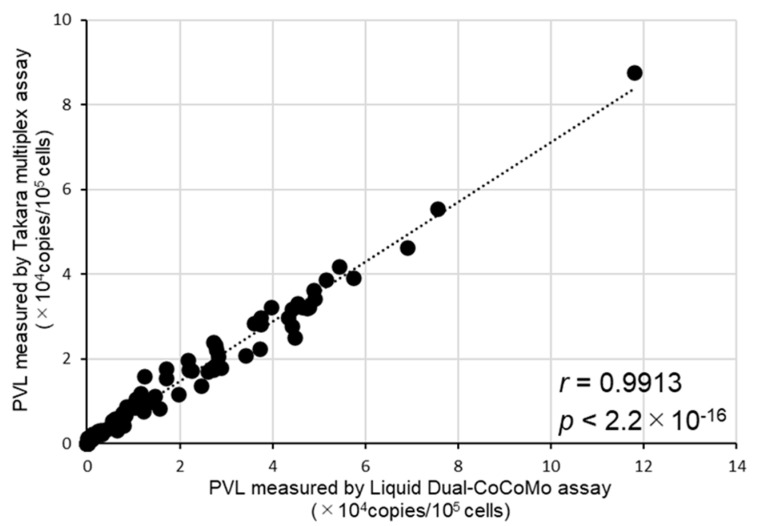
Correlation between the BLV PVLs measured by the Liquid Dual-CoCoMo and Takara multiplex assays. The PVLs of 121 cows, excluding BLV-uninfected cattle, were determined from those samples by duplicate, using the Liquid Dual-CoCoMo and Takara multiplex assays. The correlation between the PVLs measured by the Liquid Dual-CoCoMo and Takara multiplex assays was evaluated using Pearson’s correlation coefficient (*r*); *p*-values are indicated in the graphs. The dotted line represents the approximate curve.

Next, we examined the correlation between the quantitative values of PVLs measured using the Liquid Dual-CoCoMo and Takara multiplex assays. This result revealed a strong positive correlation (*r* = 0.9913; *p* = 2.2 × 10^−16^) (Figure 2), indicating that the PVL measurements reported by the Liquid Dual-CoCoMo and Takara multiplex assays agreed well. 

### 3.3. Comparison of Diagnostic Performance Between Liquid Dual-CoCoMo and Takara Multiplex Assays Using 214 Field Samples

To evaluate the diagnostic performance of the Liquid Dual-CoCoMo and Takara multiplex assays, we calculated their diagnostic sensitivity and specificity against the results of diagnosis of BLV infection by ELISA (Table 4) or Single-CoCoMo assay (Table 5) using 214 field cows. In addition, we examined the level of agreement with Cohen’s kappa coefficient between each assay and ELISA or Single-CoCoMo assay results. Firstly, by comparing the ELISA and each assay results, the Cohen’s kappa coefficient that indicated the level of agreement between the results of each assay was 0.95 (95% CI: 0.91–0.99) and 0.90 (95% CI: 0.84–0.96), respectively, which was almost perfect in both cases (Table 4). However, the Liquid Dual-CoCoMo assay showed 96.61% sensitivity (95% CI: 91.55–99.07) and 98.96% specificity (95% CI: 94.33–99.97), whereas the Takara multiplex assay showed 92.37% sensitivity (95% CI: 86.01–96.45) and 97.92% specificity (95% CI: 92.68–99.75). The results suggest that the diagnostic performance of the Liquid Dual-CoCoMo assay was higher than that of the Takara multiplex assay compared with that of ELISA. Secondly, by comparing the Single-CoCoMo assay and each assay results, the Cohen’s kappa coefficient was 0.96 (95% CI: 0.93–1.00) and 0.91 (95% CI: 0.85–0.96), respectively, which was also almost perfect in both cases (Table 5). However, the Liquid Dual-CoCoMo assay showed 97.44% sensitivity (95% CI: 92.69–99.47) and 98.97% specificity (95% CI: 94.39–99.97), whereas the Takara multiplex assay showed 93.16% sensitivity (95% CI: 86.97–97.00) and 97.94% specificity (95% CI: 92.75–99.75). Therefore, our results indicate that the diagnostic performance of the Liquid Dual-CoCoMo assay is higher than that of the Takara multiplex assay when compared to the ELISA or Single-CoCoMo assay as diagnostic reference standards.

### 3.4. Comparison of Analytical Performance Between Liquid Dual-CoCoMo and Takara Multiplex Assays

We compared the analytical sensitivity of the Liquid Dual-CoCoMo and Takara multiplex assays by detecting the BLV provirus using an infected full-length molecular clone of BLV (pBLV-IF2) (Table 6). We employed a two-fold dilution of pBLV-IF2, adjusted the proviral copy number from 25 to 0.78125 per 10^5^ cells, performed quintuplicate PCR amplifications twice, and calculated the total BLV detection percentage (Table 6). As shown in Table 6, when present at 6.25 copies or more, both methods achieved the same detection rate, successfully detecting 100% (10/10) of pBLV-IF2. At 3.125 copies, the Liquid Dual-CoCoMo and Takara multiplex assays detected 80% (8/10) and 90% (9/10) of pBLV-IF2, respectively. At 1.5625 copies, both assays detected 60% (6/10) of pBLV-IF2. When the copy number was 0.78125 copies, the Liquid Dual-CoCoMo assay detected 30% (3/10) of pBLV-IF2, whereas the Takara multiplex assay failed to detect pBLV-IF2 (0/10; 0%). The results indicate that the Liquid Dual-CoCoMo assay has a tendency to detect the BLV provirus with higher sensitivity than the Takara multiplex assay.

## 4. Discussion

In this study, we compared the diagnostic and analytical performance of the Liquid Dual-CoCoMo assay targeting the LTR region with those of the Takara multiplex assay targeting the *pol* region. First, we observed a strong positive correlation between the PVLs measured in the Liquid Dual-CoCoMo and Takara multiplex assays, indicating that the two duplex assays targeting different regions are equally useful for measuring BLV PVL in field samples. However, our results showed that the Liquid Dual-CoCoMo assay has a tendency to detect BLV provirus in field samples with higher sensitivity than that of the Takara multiplex assay. Particularly, five cows that were positive in the ELISA and Single-CoCoMo assay were positive in the Liquid Dual-CoCoMo assay but negative in the Takara multiplex assay, which may be because the CoCoMo method targets the LTR region that is present at two copies per provirus. This result is consistent with our previous finding that the CoCoMo method had higher analytical sensitivity compared with that of other methods [34]. Second, although the level of agreement between the results of each assay and those of ELISA or Single-CoCoMo assay was almost perfect in both cases, the diagnostic performance based on the results in the ELISA and Single-CoCoMo assay demonstrated that the Liquid Dual-CoCoMo assay had a higher value with diagnostic sensitivity and specificity than those of the Takara multiplex assay. Finally, the results of the analytical sensitivity analysis using the pBLV-IF2 showed that the Liquid Dual-CoCoMo assay targeting the LTR region, which is present in two copies per provirus, may detect the BLV provirus with higher sensitivity than that of the Takara multiplex assay targeting the *pol* region, which is present in a single copy per provirus, suggesting that the Liquid Dual-CoCoMo assay can be used as a diagnostic test for BLV infection. Our results indicate that this assay targeting the LTR region can be used in a global reference protocol, along with the assays targeting the *pol* and *env* regions. To the best of our knowledge, this study is the first to compare the diagnostic and analytical performances of different commercially available multiplex qPCR assays targeting different regions.

In the current study, three cows (S40, O10, and O6) were positive for BLV provirus but negative for gp51 antibodies, while two (O1 and O2) were negative for BLV provirus but positive for gp51 antibodies. These differences in the qPCR and ELISA results indicate that these cows were in the early phase of BLV infection before the antibody titer increased or that PVL was maintained at a low level, similar to our previous results [37,41] and other previous studies [51,66]. Interestingly, the two qPCR-negative but ELISA-positive cows (O1 and O2) were confirmed to have the highly polymorphic bovine lymphocyte antigen (*BoLA*)-DRB3*009:02 allele, which is a resistance allele marker associated with low BLV PVL. These results indicate that the PVL of cattle with resistant alleles may be maintained at low levels, close to the limit of detection of PCR, and at low risk of BLV transmission [39,67,68,69,70,71]. Of the three qPCR-positive but ELISA-negative cows (S40, O10, and O6), one (S40) was positive in all three qPCR assays, including the Single-CoCoMo assay, one (O6) was positive only in the Takara multiplex assay, and the other (O10) was positive only in the Single-CoCoMo assay. Including the cow (O10) that was positive only in the Single-CoCoMo assay, three cows (E1, E2, and O10) that were positive in the Single-CoCoMo assay but negative in the two multiplex qPCR assays were observed (Table 2). This result suggests that the multiplex qPCR assay may have a lower diagnostic sensitivity than that of the singleplex assay. Multiplex qPCR assay is a simple, user-friendly, and cost-effective tool that detects more than two target genes simultaneously compared with the singleplex assay [43,72]. However, the sensitivity of multiplex PCR may be lower than that of singleplex PCR owing to the self-inhibition among different sets of primers and low amplification efficiency [73]. By contrast, we also observed one cow (E3) positive in two multiplex qPCR assays but negative in the Single-CoCoMo assay (Table 2). Therefore, variability in results may be observed regardless of the method used when testing animals with low PVL. Thus, combining diagnostic methods or re-testing at intervals before the transmission risk increases is desirable for an accurate diagnosis of BLV infection.

Interestingly, our field sample results showed that one cow (O6) was negative in the ELISA, Single-CoCoMo, and Liquid Dual-CoCoMo assays, but was positive for BLV provirus detected in the Takara multiplex assay. The sample was re-tested using the Takara multiplex assay and tested positive. Recently, we reported that three types of mutations were present at very low detection rates in the nucleobase of the probe region in the LTRs of the CoCoMo method [61]. Therefore, we sequenced the partial LTR region of this sample to confirm the presence of mutations in the detection region of the CoCoMo method. The results revealed no mutations in the partial LTR region, including the probe region of the CoCoMo method, suggesting that the reason why BLV provirus was detected in only the Takara multiplex assay was not due to mutation but due to the sensitivity of the assay. Although there were no mutations in the BLV LTR target regions in this study, our previous results showed that they were present at a very low rate of 1.2% (11 of 887 samples) in the probe region of the CoCoMo method [61]. Previous studies have reported that the *pol* gene was the suitable region for the detection of BLV because methods targeting the *pol* gene had the most sensitivity compared to methods targeting the *gag* and *env* genes [44,45,47]. The World Organization for Animal Health (WOAH) Terrestrial Manual also describes a real-time PCR method targeting the *pol* gene. However, Jaworski et al. reported that the variability and nucleotide diversity of the *tax* gene were lower than those of the *pol* gene [74]; therefore, the possibility remains that mutations exist in the *pol* region as well as the LTR region. Unfortunately, we could not assess the nucleotide sequences of the Takara multiplex assay targeting the *pol* region because the nucleotide sequences of the primers of the Takara multiplex assay were not disclosed in this study. Therefore, our Liquid Dual-CoCoMo assay could be a more robust method by reconstructing new probes that consider newly reported mutations of the LTR region in future studies.

Commercially available multiplex qPCR kits for measuring BLV proviral DNA include the Liquid Dual-CoCoMo assay targeting the LTR region [43], Takara multiplex assay targeting the *pol* region [46], and SS1 BLV proviral load qPCR multiplex assay targeting the *pol* region [51]. Commercially available singleplex methods include our Single-CoCoMo assay, which targets the LTR region; the Cycleave PCR BLV detection kit, which targets the *env* region (TaKaRa Bio, Inc., Otsu, Japan), is no longer available. Using commercially available kits with standardized protocols ensures consistent results, even without skilled laboratory workers. The multiplex qPCR assay is an essential tool for the early and accurate diagnosis of BLV infection and estimation of BLV transmission risk because there is no need for the complex preparation of PCR reagents that involves preparing the target gene and host gene amplification reaction separately. Furthermore, this assay saves reagents, sample volume, laboratory equipment, and working time [43]. Moreover, it has the advantage of normalizing viral genomic DNA by simultaneously amplifying a host gene to express BLV PVLs in terms of the number of copies per cell. Therefore, these methods enable the comparison of PVLs measured across different PCR instruments and laboratories by adjusting for variations in amplification efficiency between samples [34,41]. Additionally, the Dual-CoCoMo system may simultaneously diagnose BLV infection and other retroviral infections by improving the assay to a multiplex system and adding probes and primers to detect other viruses. Previous research has reported that BLV can be a mixed infection with bovine immunodeficiency viruses (BIVs) known as bovine lentiviruses [75,76,77]. BIV also integrates into the host genome and causes persistent infection as well as BLV. BIV infection is of concern to the livestock industry due to potential damages, such as lower milk production [78,79]. BIV infection in cattle has been suspected to be associated with disease progression of BLV [78,79]. However, there are few reliable diagnostic assays for BIV infection [79]. Therefore, our developed technique should be useful for detecting other bovine retroviruses together with BLV.

We recently developed a dried reagent for the Dual-CoCoMo assay that enables PVL measurement by simply adding PCR-grade water and genomic DNA. This reagent is not only easy to handle but also simple to transport and store, making it ideal for diagnosing BLV infection in diverse regions [43]. Therefore, our Dual-CoCoMo assay targeting the LTR region can be a highly effective global protocol for quantifying the BLV PVL and diagnosing the BLV infection.

## Figures and Tables

**Table 1:** BLV detection results in ELISA, Single-CoCoMo, Liquid Dual-CoCoMo, and Takara multiplex assays.

	Methods			
	ELISA	Single-CoCoMo	Liquid Dual-CoCoMo	Takara Multiplex
Positive	118	117	115	111
Negative	96	97	99	103
Total	214	214	214	214

**Table 2:** Comparison of BLV detection results by ELISA and three qPCR assays.

Methods				Sample No./Total No. (Sampe ID)
ELISA	Single-CoCoMo	Liquid Dual-CoCoMo	TaKaRa-Multiplex	
+	+	+	+	108/214
	+	+	−	5/214
	+	−	−	2/214 (E1, E2)
	−	+	+	1/214 (E3)
	−	−	−	2/214 (O1, O2)
−	+	+	+	1/214 (S40)
	+	−	−	1/214 (O10)
	−	−	+	1/214 (O6)
	−	−	−	93/214

**Table 3:** Comparison of the detection of BLV provirus with the Liquid Dual-CoCoMo and Takara multiplex assays using 121 samples, excluding BLV-uninfected cows.

Detection of BLV Provirus		Number of Cows (Sample ID)
Liquid Dual-CoCoMo	Takara Multiplex	
+ ^a^	+	110
+	−	5
−	+	1 (O6)
−	−	5
Total		121

^a^: “+” and “–” indicate the judgement results with two assays are positive and negative, respectively.

**Table 4:** Diagnostic sensitivity, specificity, and Cohen’s kappa coefficient of agreement of Liquid Dual-CoCoMo and Takara multiplex assays compared with those of ELISA.

Methods		ELISA		Total	Sensitivity (%)	Specificity (%)	Cohen’s Kappa Coefficient ^a^
		Positive	Negative		(95% CI)	(95% CI)	(95% CI)
Liquid Dual-CoCoMo	Positive	114	1	115	96.61 (91.55–99.07)	98.96 (94.33–99.97)	0.95 (0.91–0.99)
	Negative	4	95	99			
	Total	118	96	214			
Takara-multiplex	Positive	109	2	111	92.37 (86.01–96.45)	97.92 (92.68–99.75)	0.90 (0.84–0.96)
	Negative	9	94	103			
	Total	118	96	214			

^a^ Cohen’s kappa coefficient was judged according to the following criteria proposed by Landis and Koch: <0.00 “poor agreement”; 0.00–0.20 “slight agreement”; 0.21–0.40 “fair agreement”; 0.41–0.60 “moderate agreement”; 0.61–0.80 “substantial agreement”; 0.81–1.00 “almost perfect agreement”.

**Table 5:** Diagnostic sensitivity, specificity, and Cohen’s kappa coefficient of agreement of Liquid Dual-CoCoMo and Takara multiplex assays compared with those of the Single-CoCoMo assay.

Methods		Single-CoCoMo		Total	Sensitivity (%)	Specificity (%)	Cohen’s Kappa Coefficient
		Positive	Negative		(95% CI)	(95% CI)	(95% CI)
Liquid Dual-CoCoMo	Positive	114	1	115	97.44 (92.69–99.47)	98.97 (94.39–99.97)	0.96 (0.93–1.00)
	Negative	3	96	99			
	Total	117	97	214			
Takara-multiplex	Positive	109	2	111	93.16 (86.97–97.00)	97.94 (92.75–99.75)	0.91 (0.85–0.96)
	Negative	8	95	103			
	Total	117	97	214			

**Table 6:** Comparison of BLV provirus detection frequency with Liquid Dual-CoCoMo and Takara multiplex assays.

pBLV-IF2 ^a^ (Copy Number)	BLV Provirus Detection Frequency (%)								
	Liquid Dual-CoCoMo Assay					Takara Multiplex Assay			
	Exp. 1 ^b^	Exp. 2	Total			Exp. 1	Exp. 2	Total	
25	5/5 ^c^	5/5	10/10	(100)		5/5	5/5	10/10	(100)
12.5	5/5	5/5	10/10	(100)		5/5	5/5	10/10	(100)
6.25	5/5	5/5	10/10	(100)		5/5	5/5	10/10	(100)
3.125	4/5	4/5	8/10	(80)		5/5	4/5	9/10	(90)
1.5625	3/5	3/5	6/10	(60)		2/5	4/5	6/10	(60)
0.78125	2/5	1/5	3/10	(30)		0/5	0/5	0/10	(0)
0	0/5	0/5	0/10	(0)		0/5	0/5	0/10	(0)

^a^ BLV infectious full-length molecular clone of BLV (pBLV-IF2). ^b^ Five biological replicates. ^c^ Number detected per number tested.

## Data Availability

The original contributions presented in the study are included in the article; further inquiries can be directed to the corresponding author.

## Supplementary Materials

The following supporting information can be downloaded at https://www.mdpi.com/article/10.3390/pathogens13121111/s1: Figure S1: DNA-sequence alignment of the partial BLV LTR gene region.
