# Supplementary material for: BLV-CoCoMo Dual qPCR Assay Targeting LTR Region for Quantifying Bovine Leukemia Virus: Comparison with Multiplex Real-Time qPCR Assay Targeting pol Region
Source: Pathogens. 2024 Dec 16;13(12):1111. doi: 10.3390/pathogens13121111 (PMC11677995; doi:10.3390/pathogens13121111)
Supplement: Supplementary file 1 [file pathogens-13-01111-s001.zip › pathogens-3300003-supplementary.pdf]

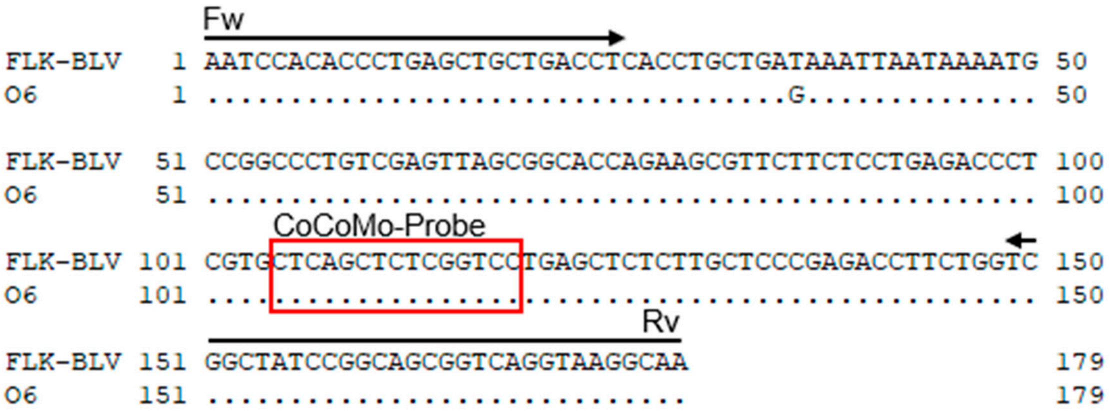

Figure S1. DNA-sequence alignment of the partial BLV LTR gene region. DNA fragments originate from a cow (O6) which was negative for the Liquid Dual-CoCoMo assay but positive for the Takara multiplex assay. The FLK-BLV sequence (EF600696) was shown as a reference at the top of the sequence alignment. The nucleotide sequences (nt) were compared with the FLK-BLV of LTR region (136 nt–314 nt). Dots indicate identity with the FLK-BLV sequence.
